# Grapevine red blotch disease: A comprehensive Q&A guide

**DOI:** 10.1371/journal.ppat.1011671

**Published:** 2023-10-12

**Authors:** Björn Krenz, Marc Fuchs, Jeremy R. Thompson

**Affiliations:** 1 German Collection of Microorganisms and Cell Cultures DSMZ GmbH, Braunschweig, Germany; 2 School of Integrative Plant Science, Plant Pathology and Plant-Microbe Biology, Cornell University, Geneva, New York, United States of America; 3 Plant Health and Environment Laboratory, Ministry for Primary Industries, Auckland, New Zealand; University of Maryland, Baltimore, UNITED STATES

## What is grapevine red blotch disease and what are its symptoms?

**Fig 1:**
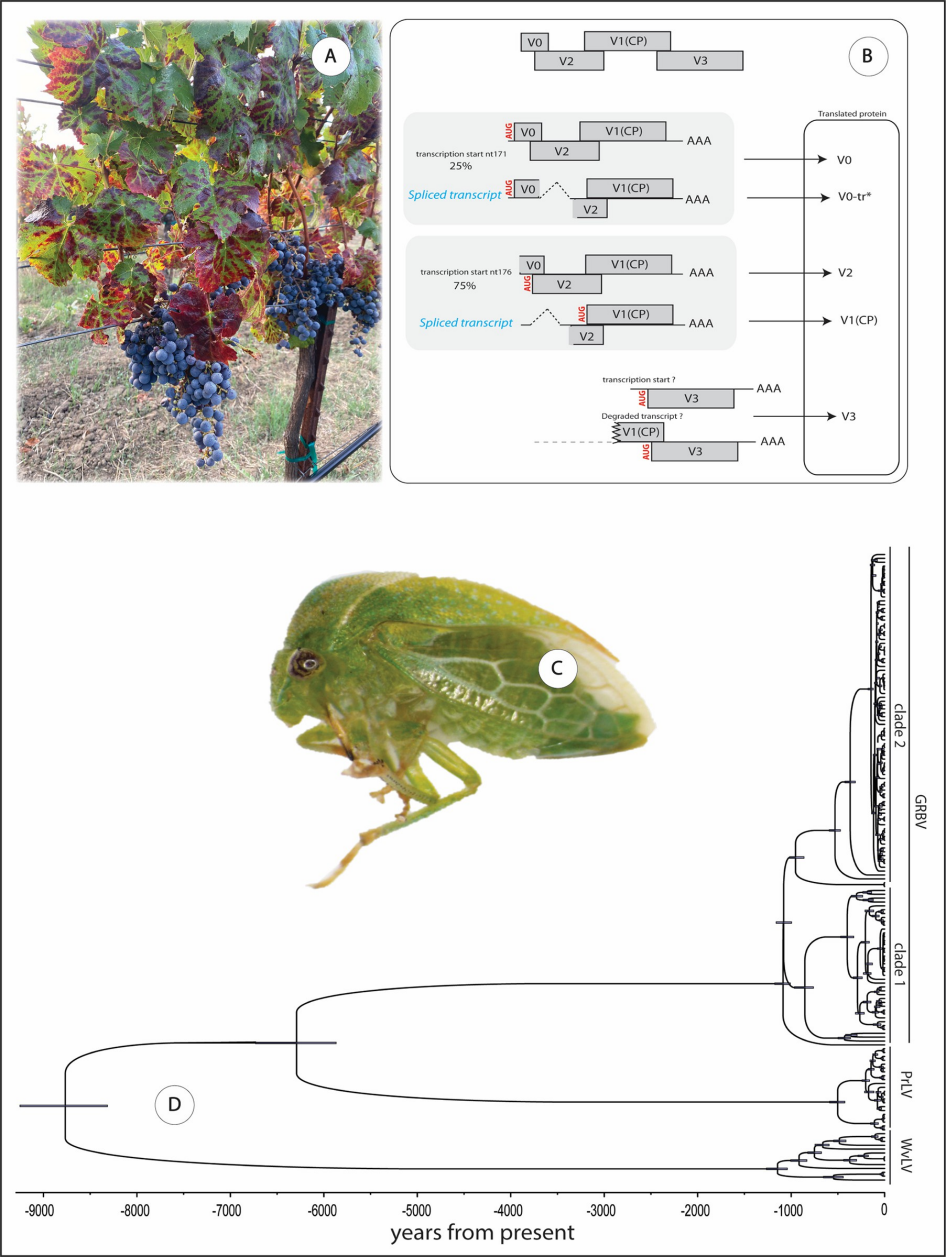
(**A**) Typical symptoms in a GRBV-infected “Cabernet franc” plant, (**B**) a model for the translational regulation of GRBV v-sense proteins [5], (**C**) the three-cornered alfalfa hopper (*Spissistilus festinus*) (courtesy of Victoria Hoyle), and (**D**) a coalescent tree for the complete genomes of 163 grabloviruses. PrLV, *Prunus* latent virus; WvLV, wild *Vitis* latent virus; horizontal bars: 95% highest posterior density (HPD) ranges for each node (shown with permission: Thompson, 2022).

Grapevine red blotch disease (GRBD) is a viral disease primarily affecting plants of *Vitis* spp. It was first described by UC Davis researchers in the mid-2000s, when they observed unusual red blotches on leaves (Fig 1A) and poor fruit ripening on “Cabernet Sauvignon” grapevines in Napa Valley, California [1]. The symptoms initially suggested a new strain of grapevine leafroll disease. Concurrently, similar symptoms were noticed in “Cabernet franc” vineyards in Lansing, New York. By 2012, a virus with a unique genome structure was identified in symptomatic grapevines [2,3], and by 2018, grapevine red blotch virus (GRBV) was confirmed as the causal agent of GRBD [4]. GRBV is taxonomically classified as a member of the *Grablovirus* genus in the Geminiviridae family. The virus has a single-stranded, circular DNA genome with 8 predicted bidirectional open reading frames (ORFs), 5 in the viral sense orientation and 3 in the complementary sense orientation. These ORFs encode proteins involved in various functions, including encapsulation of the viral genome, cell-to-cell movement, and suppression of host defenses. It is striking that GRBV also follows a splicing strategy to increase its coding capacity, with introns present in its genome, an observation relatively uncommon for geminiviruses [5–7]. Genetic analyses revealed 2 distinct phylogenetic lineages of GRBV isolates (Fig 1B) with up to 8.5% nucleotide sequence divergence [8]. Initially, the virus was thought to be confined to North America, but it has since been discovered in various countries worldwide, including the Republic of Korea, India, Switzerland, Italy, Mexico, France, Argentina, and, most recently, Australia.

## How does grapevine red blotch virus, the causative agent of GRBD, impact grapevine cultivation and the wine industry?

GRBV negatively affects grapevine cultivation and the wine industry. It impacts grape production, particularly with regard to berry quality [9]. GRBV infects a range of grape varieties, including red wine grape cultivars (e.g., Cabernet franc, Cabernet Sauvignon, Malbec, Merlot, Mourvèdre, Petit Verdot, Petite Syrah, Pinot noir, Zinfandel), white wine grape cultivars (e.g., Chardonnay, Riesling, Sauvignon blanc), interspecific hybrids, rootstocks, table grapes, and Muscadine grapes. It also infects free-living vines in northern California and southern Oregon but not in New York [10]. Symptoms in red grapevine cultivars include the eponymous red blotches and marginal reddening on the leaves. In white cultivars, foliar disease symptoms are less conspicuous, generally involving irregular chlorotic areas that may become necrotic late in the season [9,11,12]. These symptoms resemble those of viruses associated with leafroll disease. In addition to foliar symptoms, GRBV disrupts the primary and secondary metabolism of grape berry maturation by repressing ripening pathways while activating early-development metabolic routes. The virus notably reduces enzymes essential for flavonoid and anthocyanin synthesis, leading to diminished anthocyanin accumulation but increased shikimic acid and resveratrol levels. Moreover, GRBV alters key transcription factors and hormonal pathways, like the abscisic acid, ethylene, and auxin pathways, affecting normal berry ripening. Symptoms predominantly manifest post-véraison, indicating that the disease onset aligns more with the grapevine developmental stage than with viral accumulation [9,13]. GRBV threatens profitable and sustainable grape production, causing economic losses in the range of $2,231 to $68,548 (USD) per hectare over a 25-year life span of a vineyard [14].

## How is the GRBV transmitted and are there any known vectors for its transmission?

GRBV is graft-transmissible and primarily spread through infected propagative material. This tends to be the most common source of contamination in new vineyards. Over time, an increase in the number of diseased vines in certain vineyards, specifically in northern California and southern Oregon, suggests the involvement of a vector in the secondary spread of the disease [10,11,15]. However, no such spread has been reported in New York, Switzerland, France, or Italy. The three-cornered alfalfa hopper (*Spissistilus festinus*, Fig 1C) has been identified in greenhouse and vineyard experiments as a vector, transmitting GRBV from infected to healthy vines [16]. Importantly, although *S*. *festinus* is not considered a pest of grapevine, it has been shown to transmit GRBV between free-living vines, and between free-living vines and *Vitis vinifera* “Cabernet franc” and vice versa [17]. The efficiency of GRBV transmission by *S*. *festinus* seems to vary depending on the host, with greater efficiency observed with free-living vines or the experimental herbaceous host, common bean, than with *V*. *vinifera* “Cabernet franc” vines, possibly due to different host feeding behaviors of the hopper vector [17]. Furthermore, GRBV circulates within the body of the treehopper vector to be transmitted but does not use the hopper as a host, although it is transferred from one development stage through the molt to the next. Extended periods of exposure to an infected plant are necessary for *S*. *festinus* to acquire the virus; for example, 10 days exposure to an infected grapevine, the natural host, and 6 days exposure to an infected common bean were found to be required. Studies in Californian vineyards indicate that *S*. *festinus* is a vector of epidemiological relevance [16]. However, it should be noted that grapevine is not a preferred feeding host and not a reproductive host of *S*. *festinus*, suggesting opportunistic interactions between the virus, its vector, and the natural host.

## What measures are recommended for the control and prevention of GRBD in vineyards and why is it important for grapevine-growing regions to monitor and verify the presence or absence of GRBV?

Despite analyses with existing related viruses suggesting divergence from its most common recent ancestor occurred over 6,000 years ago [18], GRBV remained unnoticed in North American vineyards probably due to its symptoms being mistaken for those of leafroll viruses [2]. Given the epidemiological uncertainties of red blotch disease, the primary control measures currently advised are focused on the use of GRBV-free planting material [14]. In affected vineyards, the removal of individual diseased vines is recommended if disease incidence is less than 30%, while removing the entire vineyard is advised if disease incidence surpasses 30%. Monitoring for GRBV is vital not just for disease management at a vineyard or regional scale but also from a global perspective. Undetected GRBV can have broad implications, potentially jeopardizing vineyards across continents. For this reason, including GRBV in certification and quarantine measures is essential to ensure the health of global grapevine industries and prevent the inadvertent spread of this covert virus [14].

## What are emerging or open questions?

Despite substantial advances in understanding the biology of GRBV, several important questions about its origin and spread remain unanswered. For example, Reynard and colleagues [19] surveyed 816 *Vitis* spp. accessions from 50 countries and found GRBV in only 6 accessions, all of which originated from the United States. Similarly, Bertazzon and colleagues [20] tested 596 grapevine samples in Northern Italy and found only 2 accessions infected with GRBV, “Queen” of American origin and Italian “Incrocio Dalmasso VIII-5,” the latter’s infection source remaining a mystery. Hence, it seems likely that GRBV originated in North America and spread globally via the unintentional movement of infected cuttings. Evidence from symptomatic, GRBV-infected leaves collected in Sonoma County, California in 1940 supports this hypothesis [21].

Despite these findings, several open questions warrant further investigation:

1. How widespread is the disease? There are concerns about potential spread wherever grapes are grown, especially if propagative material was imported from California between 1920 and 2015.
2. How does GRBV damage grapevines?
3. Is GRBV the only causal agent of the disease? Grapevine diseases are typically caused by multiple viruses, raising the question of whether other viruses could be involved in GRBD—such as wild *Vitis* latent virus (WvLV).
4. Are there other vectors of the disease? This is a subject of ongoing research [22].
5. How much time is required for a vector to acquire the virus?
6. How long does the virus persist inside a vector? Preliminary experiments suggest that the virus persists for at least 30 days within *S*. *festinus*.
7. How long does it take for a grapevine exposed to a viruliferous *S*. *festinus* to display disease symptoms?
8. What are some refuge plants in a vineyard ecosystem, if *V*. *vinifera* is not a preferred feeding and reproductive host?
9. What patterns can be observed in the vector’s movement across vineyard ecosystems?
10. What is the biological significance of the 2 phylogenetic clades of GRBV, given that isolates of both clades are etiological agents of GRBD, cause similar disease symptoms, and are transmitted by *S*. *festinus*?
11. Are there ways to reduce the disease’s impact beyond roguing, given that other cultural responses have yet to prove satisfactory?
12. Can faster, less costly tests be developed for identifying the disease’s presence in vineyards? Technologies such as LAMP [23] are currently being used in vineyards in Northern California, but further advancements could enhance disease detection and management.

These unanswered questions underscore the intricate nature of GRBD and the challenges faced in its prevention and management. Particularly pressing are concerns regarding the true extent of the disease’s spread, especially given the historical contribution of infected planting stocks in primary infection, the existence of GRBV vectors other than *S*. *festinus*, and the potential involvement of other viruses in GRBD’s causation. Furthermore, the quest for more efficient virus detection methods, such as advanced versions of LAMP, highlights the industry’s need for swift and cost-effective diagnostic measures. Continued research is paramount to address these critical inquiries and ensure the sustainability and health of global grapevine industries.
